# Supplementary material for: RNA-seq analysis of the human surfactant air-liquid interface culture reveals alveolar type II cell-like transcriptome
Source: Mol Ther Methods Clin Dev. 2021 Nov 24;24:62–70. doi: 10.1016/j.omtm.2021.11.006 (PMC8688965; doi:10.1016/j.omtm.2021.11.006)
Supplement: Document S1. Figures S1–S5 and Tables S1 and S2 [file mmc1.pdf]

OMTM, Volume *24*

Supplemental information

RNA-seq analysis of the human surfactant air-liquid interface culture reveals alveolar type II cell-like transcriptome

Altar M. Munis, Benjamin Wright, Frederic Jackson, Helen Lockstone, Stephen C. Hyde, Catherine M. Green, and Deborah R. Gill

Supplementary Materials

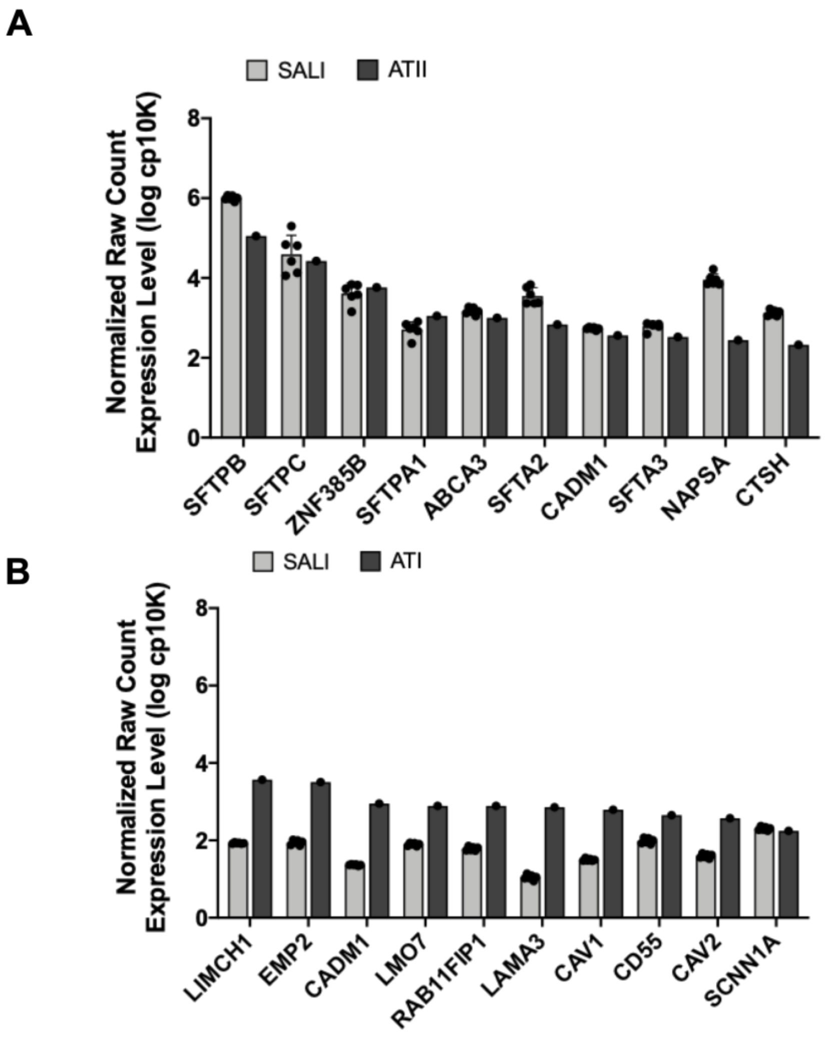

Figure S1: Comparison of the average of normalized count values of select ATII and ATI markers in SALI cultures versus primary cells. Bar graphs depicting the average normalized count values (in log cp10K) of selected **(A)** ATII and **(B)** ATI markers. Each data point represents an individual wt SALI library. The data for the primary human ATII and ATI cells were obtained from the Genotype-Tissue Expression (GTEx) portal on 26/10/2021. The GTEx was supported by the Common Fund of the Office of the Director of the National Institutes of Health, and by NCI, NHGRI, NHLBI, NIDA, NIMH, and NINDS.

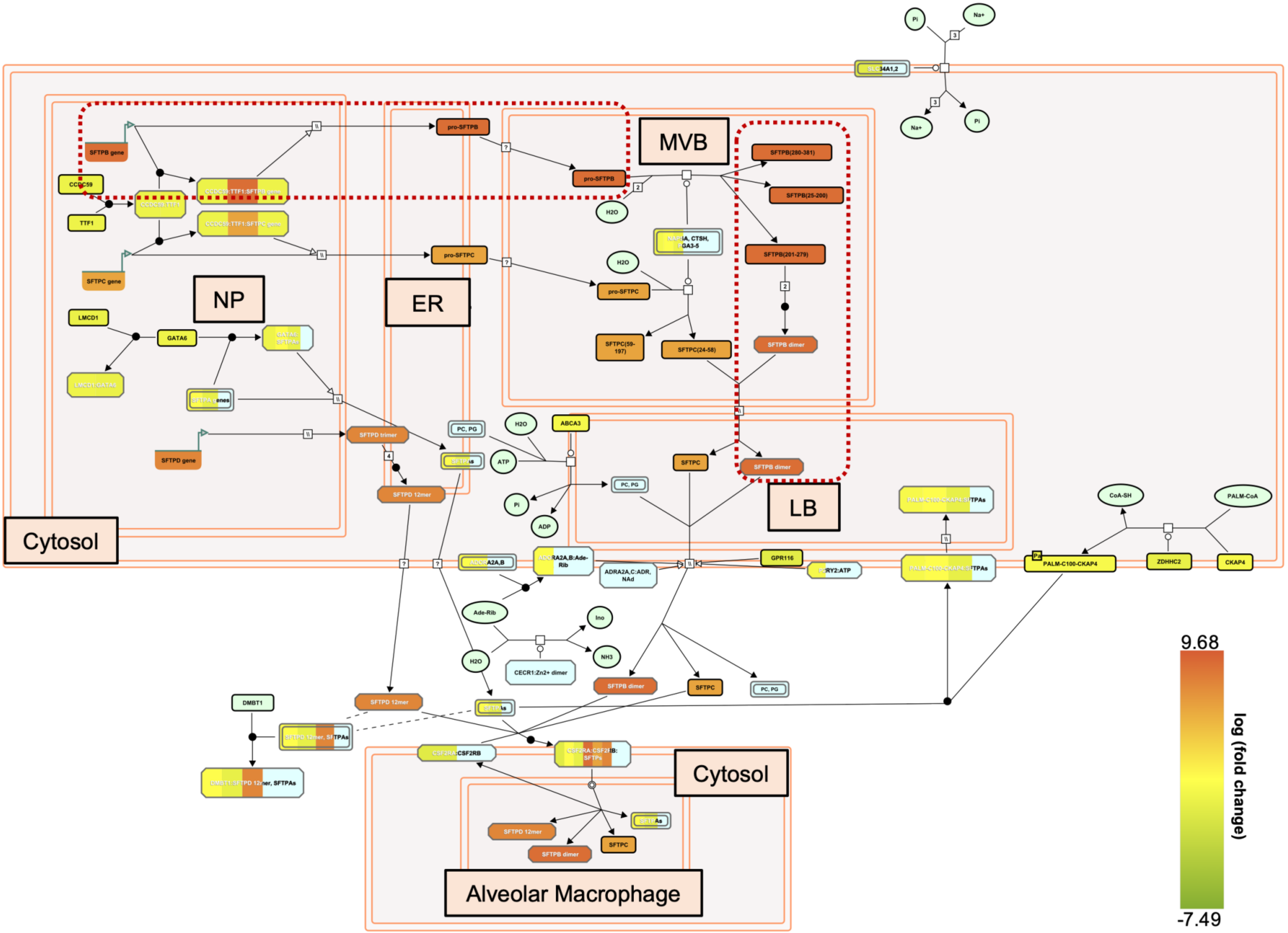

MVB
NP
ER
LB
Cytosol
Alveolar Macrophage
SFTPB gene
SFTPC gene
SFTPD gene
CCDC59
TTF1
LMCD1
GATA6
pro-SFTPB
pro-SFTPC
SFTPB(280-381)
SFTPB(25-200)
SFTPB(201-279)
SFTPB dimer
SFTPC(24-58)
SFTPD trimer
SFTPD 12mer
SFTPC
PC, PG
H2O
ATP
ADP
Pi
Na+
ABCA3
GPR116
DMBT1
Ade-Rib
Ino
NH3
CECR1:Zn2+ dimer
ADRA2A,C:ADR, NAd
CSF2RA:CSF2RB
PALM-C100-CKAP4
ZDHHC2
CKAP4
CoA-SH
PALM-CoA
9.68
-7.49
log (fold change)

Figure S2: A diagram of the surfactant metabolism pathway depicting the relative expression levels of the genes and proteins involved in SALI culture. The differential gene expression data of SALI culture versus submerged H441 cells were analyzed and the results were overlaid on the surfactant metabolism pathway using the open-source pathway database Reactome [1-3]. Regulation of all genes and proteins are labelled and colored based on log (fold change) summarized in the legend. Cellular compartments are labelled. Green circles represent the chemical co-factors that are involved in the pathway. NP: nucleoplasm; ER: endoplasmic reticulum; MVB: multivesicular body; LB: lamellar body; Pi: inorganic phosphate; Na^+: sodium ion; ATP: adenosine triphosphate; ADP: adenosine diphosphate; CoA-SH: coenzyme A; PALM-CoA: Paralemmin-coenzyme A complex; H_2O: water; Ade-Rib: extracellular adenosine; Ino: inosine; NH3: ammonia.

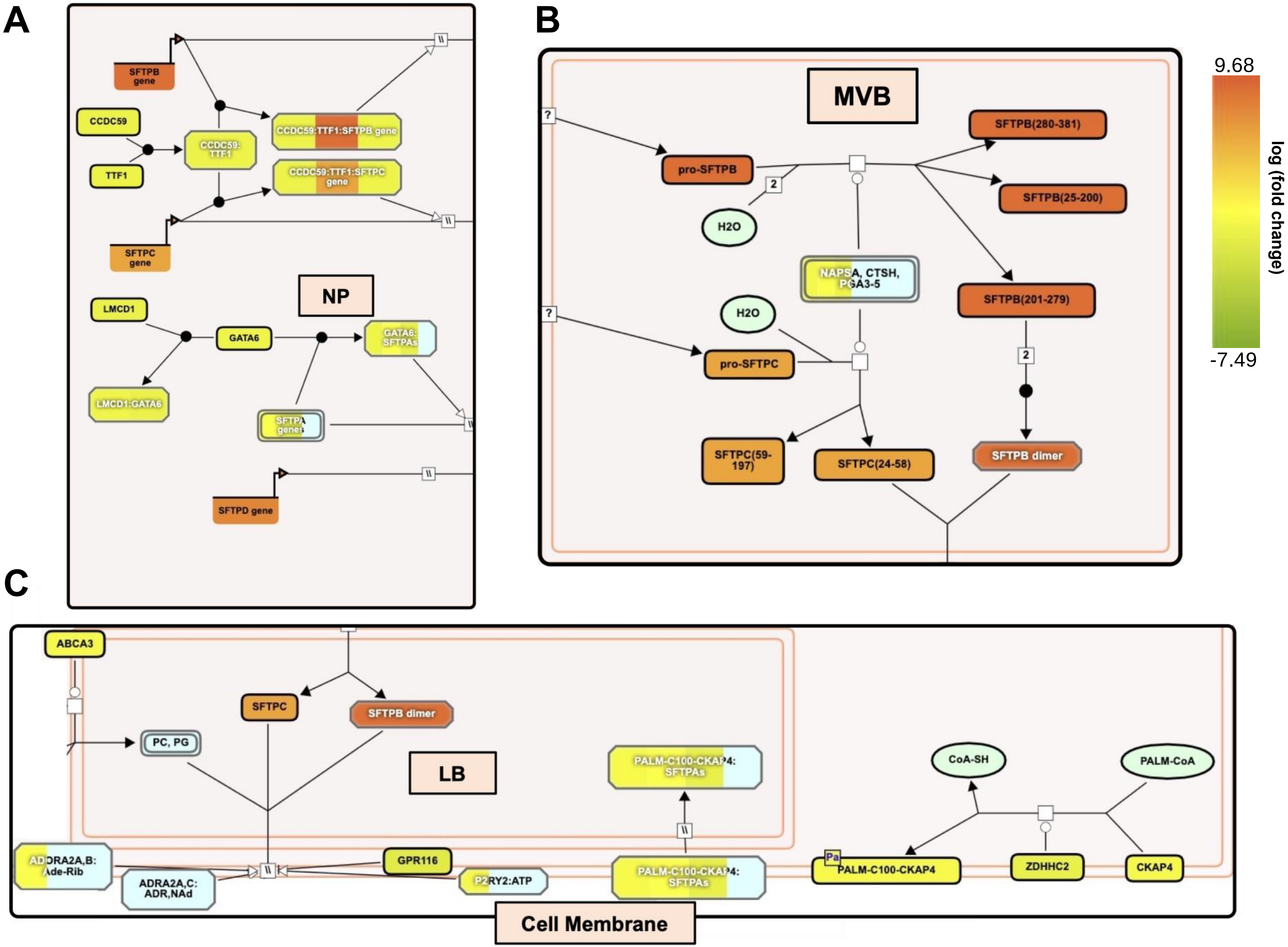
A
SFTPB gene
CCDC59
TTF1
CCDC59: TTF1
CCDC59:TTF1:SFTPB gene
CCDC59:TTF1:SFTPC gene
SFTPC gene
NP
LMCD1
GATA6
GATA6: SFTPAs
LMCD1:GATA6
SFTPA genes
SFTPD gene
B
MVB
?
pro-SFTPB
2
H2O
NAPSA, CTSH, PGA3-5
SFTPB(280-381)
SFTPB(25-200)
SFTPB(201-279)
pro-SFTPC
SFTPC(59-197)
SFTPC(24-58)
SFTPB dimer
9.68
-7.49
log (fold change)
C
ABCA3
PC, PG
SFTPC
SFTPB dimer
LB
PALM-C100-CKAP4: SFTPAs
CoA-SH
PALM-CoA
ADRA2A,B: Ade-Rib
ADRA2A,C: ADR,NAd
GPR116
P2RY2:ATP
Pa
PALM-C100-CKAP4
ZDHHC2
CKAP4
Cell Membrane

Figure S3: Genes involved in lung surfactant metabolism are upregulated in SALI cultures. Diagrams of the cellular compartments **(A)** nucleoplasm, **(B)** multivesicular bodies, and **(C)** lamellar bodies depicting the relative expression levels of the surfactant metabolism genes and proteins in SALI culture. The differential gene expression data of SALI culture versus submerged H441 cells were analyzed and the results were overlaid on the surfactant metabolism pathway using the open-source pathway database Reactome [1-3] (See also *Figure S1)*. Regulation of all genes and proteins are labelled and are colored based on log (fold change), depicted in the legend. Cellular compartments are labelled. Green circles indicate the chemical co-factors that are involved in the pathway. Light blue color indicated genes and/or proteins that have not been detected in the SALI culture. NP: nucleoplasm; ER: endoplasmic reticulum; MVB: multivesicular body; LB: lamellar body; Pi: inorganic phosphate; Na^+: sodium ion; ATP: adenosine triphosphate; ADP: adenosine diphosphate; CoA-SH: coenzyme A; PALM-CoA: Paralemmin-coenzyme A complex; H_2O: water; Ade-Rib: extracellular adenosine; Ino: inosine; NH_3: ammonia.

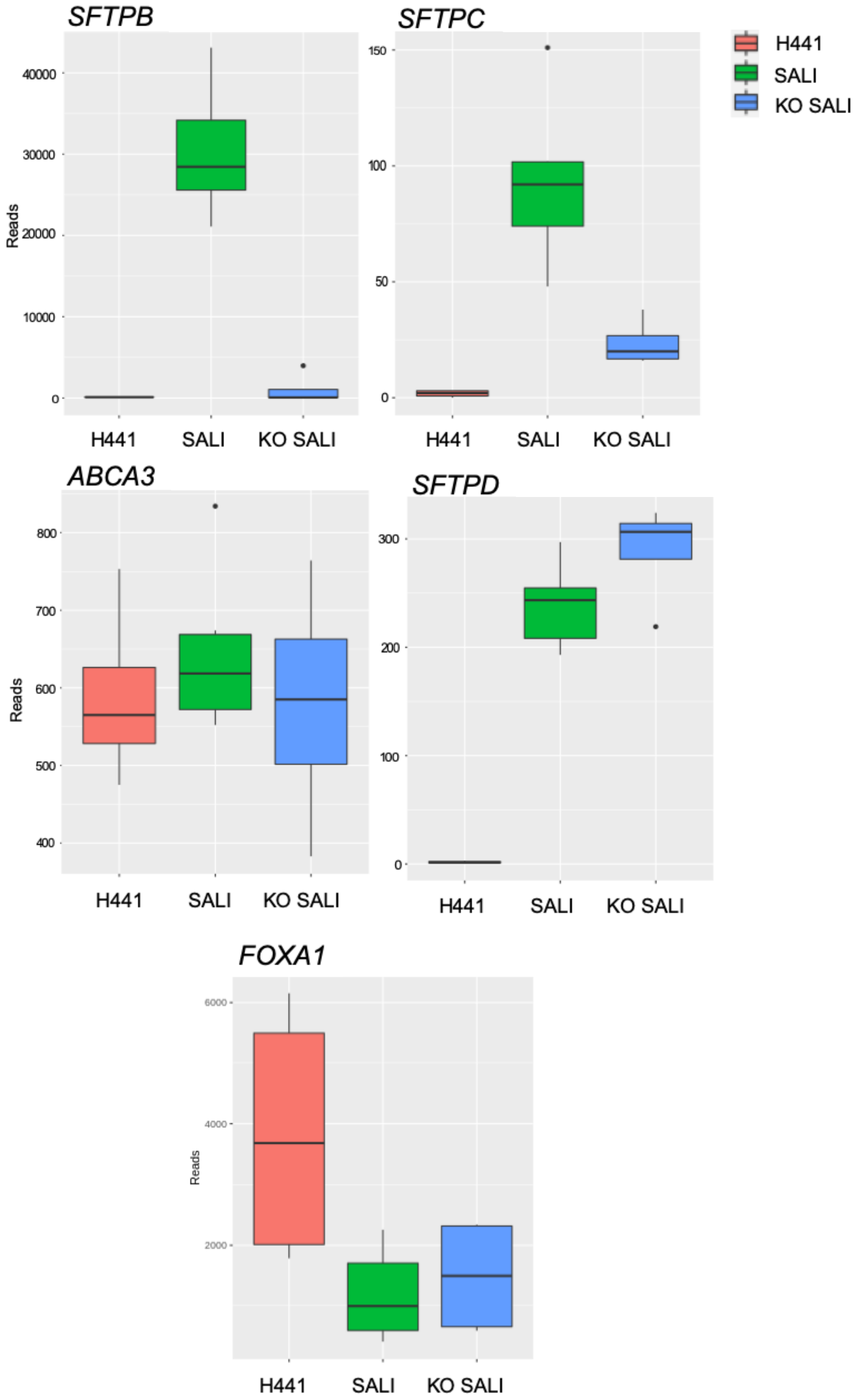

Figure S4: Box and whisker plot depicting the expression levels of *SFTPB*, *SFTPC*, *ABCA3*, *SFTPD,* and *FOXA1* in H441 cells, wild-type and *SFTPB* KO SALI

cultures. Reads were aligned to the human reference genome (GRCh37) using HISAT2[4] and duplicate reads removed using the Picard 'MarkDuplicates' tool (http://broadinstitute.github.io/picard). Reads mapping uniquely to Ensembl-annotated genes were summarized using featureCounts[5]. The raw gene count matrix was imported into the R/BioConductor environment[6,7] for further processing and analysis with the edgeR package[8,9].

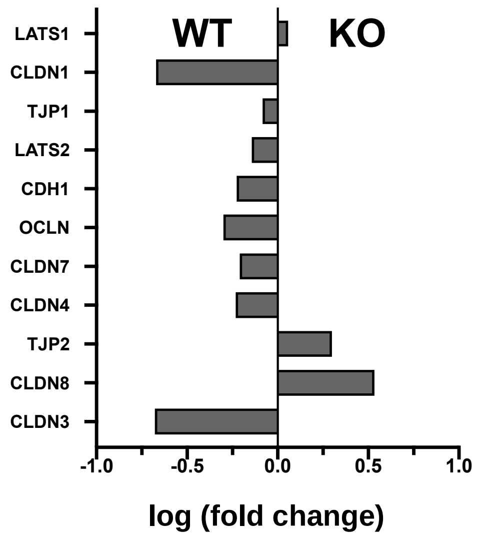

Figure S5: Differential expression analysis of the major tight junction proteins in *SFTPB* KO versus wt SALI cultures. The bar graph represents the mean differential expression of several key tight junction proteins in wt SALI cultures compared with *SFTPB* KO SALI cultures. The log (fold change) values were calculated via the bioinformatics analyses described in the methods section.

Supplementary Table 1: The list of TaqMan gene expression assays used in this study.

**Target**	**TaqMan Gene Expression Assay ThermoFisher ID#**
SFTPB	Hs00167036_m1
SFTPC	Hs00161628_m1
SFTPD	Hs01108490_m1
EEA1	Hs00929215_m1
STX2	Hs00984922_m1
LAMP3	Hs01111316_m1
SNAP23	Hs00187075_m1
ABCA3	Hs00184543_m1
(EIF2AK3) PERK	Hs00984003_m1
ATF4	Hs00909569_g1
DDIT3	Hs01090850_m1
HSPA1A	Hs00359163_s1
HSPA5	Hs99999174_m1
PIK3C3	Hs00176908_m1
PDIA3	Hs00607126_m1
TRAF2	Hs00184192_m1
XBP1	Hs00231936_m1
CASP3	Hs00234387_m1
CASP9	Hs00609647_m1
BAX	Hs00180269_m1
PDPN	Hs00366766_m1
CAV1	Hs00971716_m1
AGER	Hs00153957_m1
HOPX	Hs04188695_m1
ACTB	Hs99999903_m1

Supplementary Table 2: The list of antibodies used in this study.

Target	Antibody	Catalogue Number (Company)
ABCA3	Rabbit anti-ABCA3 polyclonal antibody	#PA5-52478 (Invitrogen)
EEA1	Rabbit anti-EEA1 polyclonal antibody	#ab2900 (Abcam)
STX2	Rabbit anti-STX2 polyclonal antiserum	#110 022 (Synaptic Systems)
β-tubulin	Rabbit anti-beta tubulin polyclonal antibody	#ab6046 (Abcam)
PERK	Rabbit anti-PERK monoclonal antibody	#C33E10 (Cell Signaling Technology)
HSPA5 (BiP)	Rabbit anti- GRP78 BiP polyclonal antibody	#ab21685 (Abcam)
GAPDH	Rabbit anti-GAPDH polyclonal antibody	#G9545 (Sigma)
SPB	Rabbit anti-Pro + mature SPB polyclonal antibody	#ab40876 (Abcam)
Anti-rabbit IgG	Swine anti-rabbit IgG, HRP conjugated	#P0399 (Dako)
Anti-rabbit IgG	Donkey anti-rabbit IgG, Alexa-Fluor 594 conjugated	#A21207 (Invitrogen)

REFERENCES

1. Croft, D., Mundo, A.F., Haw, R., Milacic, M., Weiser, J., Wu, G., Caudy, M., Garapati, P., Gillespie, M., Kamdar, M.R., et al. (2014). The Reactome pathway knowledgebase. Nucleic Acids Res *42*, D472-477. 10.1093/nar/gkt1102.
2. Fabregat, A., Jupe, S., Matthews, L., Sidiropoulos, K., Gillespie, M., Garapati, P., Haw, R., Jassal, B., Korninger, F., May, B., et al. (2018). The Reactome Pathway Knowledgebase. Nucleic Acids Res *46*, D649-D655. 10.1093/nar/gkx1132.
3. Fabregat, A., Sidiropoulos, K., Garapati, P., Gillespie, M., Hausmann, K., Haw, R., Jassal, B., Jupe, S., Korninger, F., McKay, S., et al. (2016). The Reactome pathway Knowledgebase. Nucleic Acids Res *44*, D481-487. 10.1093/nar/gkv1351.
4. Kim, D., Langmead, B., and Salzberg, S.L. (2015). HISAT: a fast spliced aligner with low memory requirements. Nat Methods *12*, 357-360. 10.1038/nmeth.3317.
5. Liao, Y., Smyth, G.K., and Shi, W. (2014). featureCounts: an efficient general purpose program for assigning sequence reads to genomic features. Bioinformatics *30*, 923-930. 10.1093/bioinformatics/btt656.

6. Team, R.C. (2021). R: A language and environment for statistical computing. https://www.R-project.org/.
7. Huber, W., Carey, V.J., Gentleman, R., Anders, S., Carlson, M., Carvalho, B.S., Bravo, H.C., Davis, S., Gatto, L., Girke, T., et al. (2015). Orchestrating high-throughput genomic analysis with Bioconductor. Nat Methods *12*, 115-121. 10.1038/nmeth.3252.
8. Robinson, M.D., McCarthy, D.J., and Smyth, G.K. (2010). edgeR: a Bioconductor package for differential expression analysis of digital gene expression data. Bioinformatics *26*, 139-140. 10.1093/bioinformatics/btp616.
9. McCarthy, D.J., Chen, Y., and Smyth, G.K. (2012). Differential expression analysis of multifactor RNA-Seq experiments with respect to biological variation. Nucleic Acids Res *40*, 4288-4297. 10.1093/nar/gks042.
