# Supplementary material for: RNA-seq analysis of the human surfactant air-liquid interface culture reveals alveolar type II cell-like transcriptome
Source: Mol Ther Methods Clin Dev. 2021 Nov 24;24:62–70. doi: 10.1016/j.omtm.2021.11.006 (PMC8688965; doi:10.1016/j.omtm.2021.11.006)
Supplement: Document S2. Article plus supplemental information [file mmc2.pdf]

Molecular Therapy
Methods & Clinical Development
Original Article

RNA-seq analysis of the human surfactant air-liquid interface culture reveals alveolar type II cell-like transcriptome

Altar M. Munis,[1] Benjamin Wright,[2] Frederic Jackson,[3] Helen Lockstone,[2] Stephen C. Hyde,[1] Catherine M. Green,[3,4] and Deborah R. Gill[1]

[1]Gene Medicine Group, Nuffield Division of Clinical Laboratory Sciences, Radcliffe Department of Medicine, University of Oxford, Oxford OX3 9DU, UK; [2]Bioinformatics and Statistical Genetics Core, Wellcome Trust Center for Human Genetics, University of Oxford, Oxford OX3 7BN, UK; [3]Clinical BioManufacturing Facility, Nuffield Department of Medicine, University of Oxford, Oxford OX3 7JT, UK; [4]Chromosome Dynamics, The Wellcome Center for Human Genetics, University of Oxford, Oxford OX3 7BN, UK

Understanding pulmonary diseases requires robust culture models that are reproducible, sustainable in long-term culture, physiologically relevant, and suitable for assessment of therapeutic interventions. Primary human lung cells are physiologically relevant but cannot be cultured *in vitro* long term and, although engineered organoids are an attractive choice, they do not phenotypically recapitulate the lung parenchyma; overall, these models do not allow for the generation of reliable disease models. Recently, we described a new cell culture platform based on H441 cells that are grown at the air-liquid interface to produce the SALI culture model, for studying and correcting the rare interstitial lung disease surfactant protein B (SPB) deficiency. Here, we report the characterization of the effects of SALI culture conditions on the transcriptional profile of the constituent H441 cells. We further analyze the transcriptomics of the model in the context of surfactant metabolism and the disease phenotype through *SFTPB* knockout SALI cultures. By comparing the gene expression profile of SALI cultures with that of human lung parenchyma obtained via single-cell RNA sequencing, we found that SALI cultures are remarkably similar to human alveolar type II cells, implying clinical relevance of the SALI culture platform as a non-diseased human lung alveolar cell model.

INTRODUCTION

Interstitial lung diseases (ILDs) comprise a variety of disorders broadly related to the lung interstitium that are characterized by inflammation and buildup of scar tissue in the lungs.[1] Affecting the alveolar epithelium, these disorders lead to abnormalities in gas exchange, decreased lung capacity, and, in many cases, including surfactant disorders, respiratory failure, and loss of life. ILDs such as idiopathic pulmonary fibrosis and surfactant-related pulmonary alveolar proteinosis are very severe with mean survival measured in months or years.[2,3] While environmental exposure and systemic diseases contribute, surfactant-associated dysfunction disorders are most commonly caused by genetic variants leaving lung transplantation and gene therapy/editing approaches as the only options for curative interventions.[4]

Further understanding the mechanistic outcomes of the disorders, as well as screening potential drug and treatment approaches, requires robust models of ILDs, based on the alveolar epithelium comprising alveolar type II (ATII) and I (ATI) cells. As primary human alveolar cells lose their functional characteristics when cultured *ex vivo*,[5,6] a number of approaches have been undertaken to develop such pulmonary models *in vitro*. A promising approach was the establishment of organoid-like spheres from isolated primary human ATII cells or stem cells,[7] but these alveolospheres failed to replicate the structure of the alveolus, lacking cells expressing markers of ATI cells. Similarly, in the case of proximal lung bud organoids, these were phenotypically closer to a developing fetal lung.[8–10] Finally, these models do not easily allow for systematic manipulation of cells to reliably generate successful disease models, as they cannot be reliably expanded in culture following CRISPR-Cas9-based interventions.

Recently, we described a new *in vitro* cell culture model that replicated key characteristics of human ATII cells and was suitable for study of ILDs, such as surfactant protein B (SPB) deficiency.[11] The human surfactant air-liquid interface (SALI) culture, based on the human H441 adenocarcinoma cell line, has advantages over other alveolar models, such as primary ATII cells and organoids, as it is both reproducible and sustainable in long-term culture, suitable for high-throughput screening of therapeutic interventions, and also allows for establishment of disease models through genetic modifications. The SALI cultures successfully recapitulated key characteristics of primary ATII cells, demonstrated by RNA and protein expression of markers, and

Received 12 April 2021; accepted 19 November 2021;
https://doi.org/10.1016/j.omtm.2021.11.006.

Correspondence: Deborah R. Gill, PhD, Gene Medicine Group, Nuffield Division of Clinical Laboratory Sciences, Radcliffe Department of Medicine, University of Oxford, Oxford OX3 9DU, UK.
E-mail: deborah.gill@ndcls.ox.ac.uk

 © 2021 The Authors.
This is an open access article under the CC BY license (http://creativecommons.org/licenses/by/4.0/).

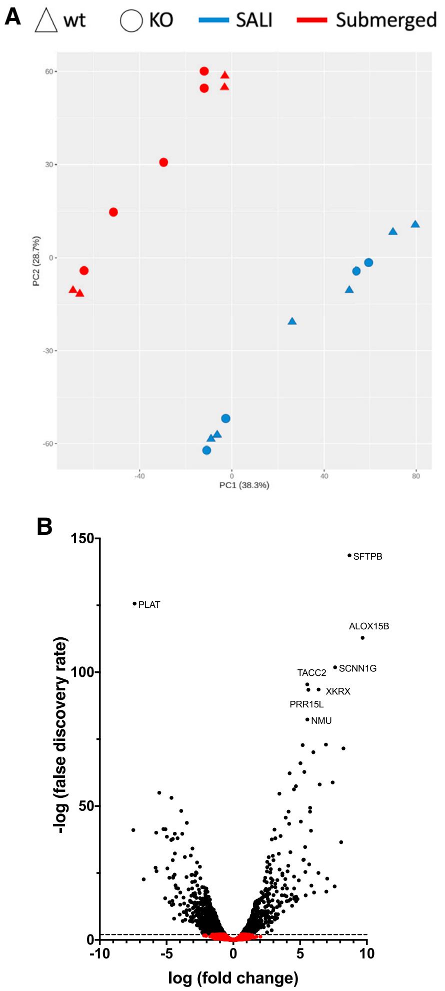

Figure 1. The SALI culture condition is the stronger driver for transcriptomic changes

(A) Principal-component analysis (PCA) plot of wild-type (WT) and knockout (KO) submerged H441 and SALI culture samples. The clustering of samples indicates that the culture conditions have the strongest impact on differential gene expression. Each data point indicates an independent replicate. (B) Volcano plot comparing the differential gene expression in SALI cultures versus submerged H441 cells. Differentially expressed genes with false discovery rates (using Benjamini-Hochberg adjusted p values) less than 0.01 are represented with black dots. The dotted line indicates –log (false discovery rate) = 2. The top 8 genes are labeled.

demonstrated functional barrier properties. We used this new culture system to show that gene therapy can be utilized *in vitro* to successfully replace the deficient SPB in the *SFTPB* knockout (KO) SALI model.

Here, we explore the transcriptomic profiles of the wild-type (WT) and KO SALI culture models to assess how this platform relates to the primary human alveolar epithelium and how the gene expression profiles of H441 cells respond to the different culturing conditions. Through RNA sequencing (RNA-seq) analyses, we show that the SALI culture condition significantly alters the transcriptional profile of H441 cells, especially of the genes relating to the surfactant metabolism. We further compare the transcriptome of the SALI cultures to that of lung alveolar epithelium determined via single-cell RNA-seq (scRNA-seq).[12] Finally, we investigate the transcriptomic consequences of the *SFTPB* KO on SALI cultures and how they correlate to the phenotypic changes we previously observed.

RESULTS

The SALI cultures transcriptome replicates that of primary ATII cells

In our previous study,[11] we demonstrated a robust physiological correlation between human SALI cultures and primary ATII cells, supported by RNA and protein expression of cell-type-specific markers. We aimed to further elaborate on this finding and determine whether the new SALI culture platform could constitute a clinically relevant model of the alveolar epithelium in a non-diseased lung. For this, independent replicates of WT and *SFTPB* KO H441 cells were grown in submerged or SALI conditions, comprising four sample types. Total RNA was extracted and enriched for poly(A)-containing mRNAs, after which a cDNA library was prepared, multiplexed, and sequenced using the Illumina NextSeq platform.

Global transcriptome analysis of the four different cultures clearly demonstrated that the SALI culture condition was the major driver of the transcriptional changes in H441 cells (Figure 1A). Conversely, the *SFTPB* KO culture samples clustered closely with their respective WT counterparts implying a targeted, as opposed to, systemic dysregulation of gene expression pathways. Differential gene expression analysis of SALI cultures versus submerged H441 cells revealed that a total of 2,242 genes were significantly ($p < 0.01$) altered (956 upregulated, 1,286 downregulated) between the two culture conditions (Figure 1B). Strikingly, *SFTPB* was the most significantly upregulated gene in SALI cultures, further affirming the suitability of SALI cultures to model SPB deficiency.

We further aimed to assess the physiological relevance of the distinct transcriptomic signatures of the submerged H441 cells and SALI cultures. A gene set enrichment analysis was performed to determine whether the RNA-seq data could recapitulate gene expression signatures associated with the primary alveolar epithelium. We, therefore, compared the transcriptional profile of the SALI culture with datasets created by Reyfman et al. via scRNA-seq of healthy human lung samples.[12] As it is reported that H441 cells are derived from both ATII and club cell lineages,[13–15] we included club cell-specific data in our analysis along with the two major alveolar cell types: ATI and ATII. When the expression levels of the top 50 cell-specific genes were interrogated in SALI cultures, a significant ($p < 0.01$) upregulation of the majority of ATII cell-specific genes was observed (Figure 2A). In contrast, no such clear trend was present for either ATI or club cell-specific genes (Figures 2B and 2C). Comparison of the average of normalized count values of these markers in SALI culture versus the primary human cells revealed similar levels of RNA expression for ATII markers but lower levels of ATI markers (Figure S1, data

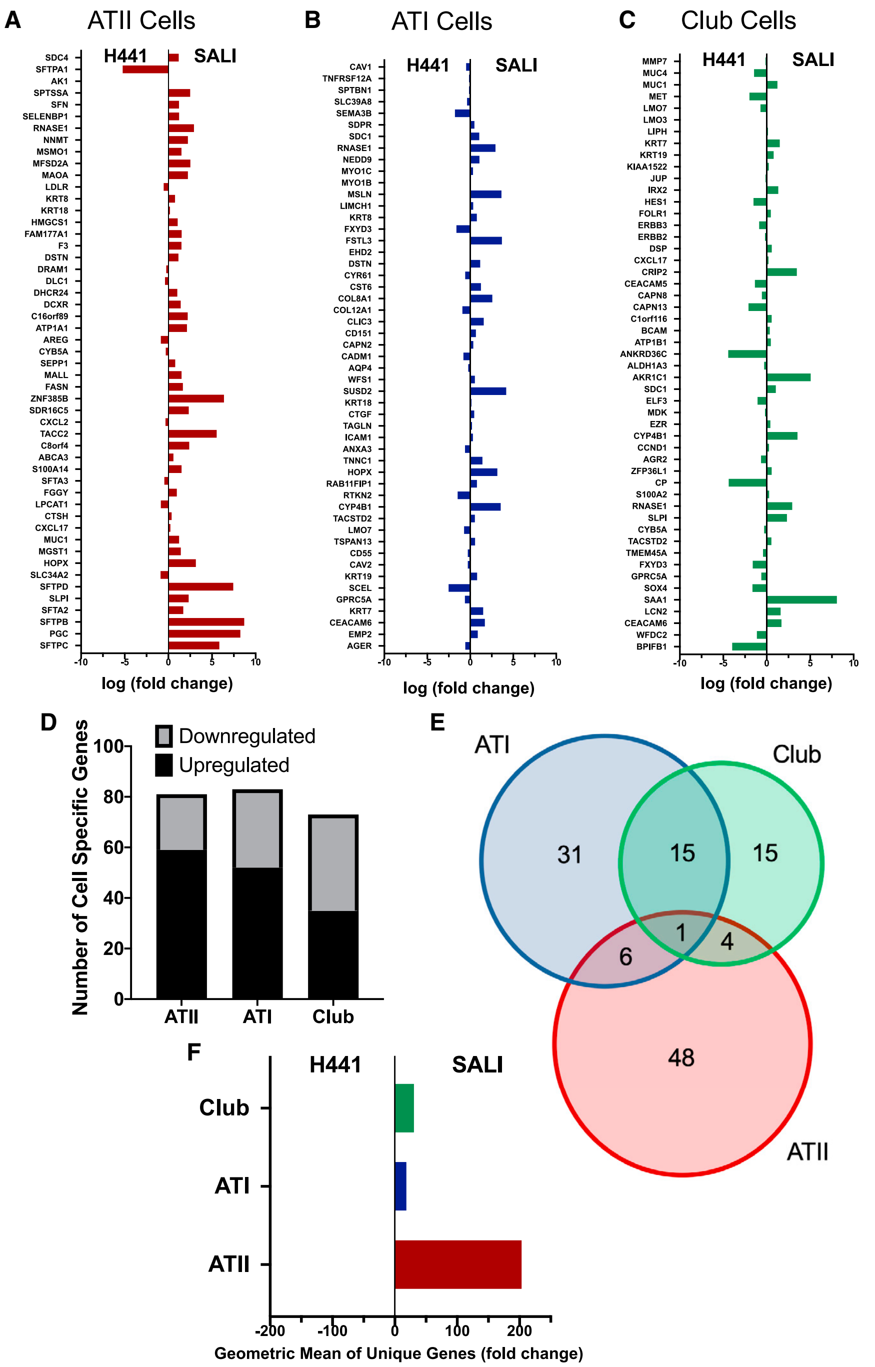

Figure 2. The SALI culture transcriptome matches that of ATII cells isolated from healthy human lung

The top 50 genes expressed in primary, human ATII, ATI, and club cells were curated based on previously published scRNA-seq experiments of healthy human lungs.[12] The bar graphs represent the mean differential expression of these (A) ATII, (B) ATI, and (C) club cell-specific genes in SALI cultures compared with submerged H441 cultures. (D) Expression regulation breakdown of the top 100 cell-type-specific genes, identified by Reyfman et al.,[12] in SALI cultures. (E) All upregulated genes identified in (D) were plotted on a Venn diagram depicting their distribution among the three cell types. (F) The genes unique for each cell type, highlighted in the Venn diagram, were identified and their overall expression in SALI cultures versus submerged H441 cultures plotted in a bar graph.

not shown). We further investigated the transcriptomic regulation of the top 100 cell-specific genes (Figure 2D). While similar numbers of genes from each dataset were detected in SALI cultures, >70% of ATII cell-related genes were found to be upregulated. In contrast, for ATI and club cell-specific genes this number was 62% and 48%, respectively. Furthermore, while a considerable number of the upregulated genes were shared between ATI and club cell datasets, >80% of the ATII cell markers were unique (Figure 2E). Further differential gene expression analysis of all unique upregulated markers within each dataset clearly demonstrated significant ($p < 0.01$) enrichment of ATII cell genes in SALI cultures (Figure 2F). These findings are consistent with our previous conclusions, showing the capacity for SALI cultures to recapitulate lung parenchyma and, in addition, highlight the striking similarities between the transcriptome of SALI cultures and the gene expression dataset acquired from primary human ATII cells.[12]

Surfactant metabolism pathway is upregulated in SALI cultures

The SALI culture was originally developed to study SPB deficiency, as a first step to assess gene therapy and gene editing interventions, but can be extended to other monogenic ILDs relating to ATII cells, specifically to surfactant metabolism. We, therefore, evaluated the differential gene expression data of SALI versus submerged H441 cultures focusing on the surfactant metabolism pathway unique to ATII cells (Figure S2). As expected from our previous report, we observed considerable upregulation in the genes involved in the production and processing of SPB (Figure S3). In parallel with the enrichment of ATII-specific genes, the majority of the genes involved in the surfactant metabolism pathway was highly expressed in SALI cultures. For example, significant upregulation of *SFTPC* and *SFTPD* genes was observed (Figures S3A and S4). Furthermore, the enzymes (e.g., *NAPSA* and *CTSH*) that are responsible for the correct processing of both SPB and SPC in multivesicular bodies were detected (Figure S3B), as also demonstrated by protein expression of SPB intermediates and mature dimers in SALI cultures as we reported previously.[11]

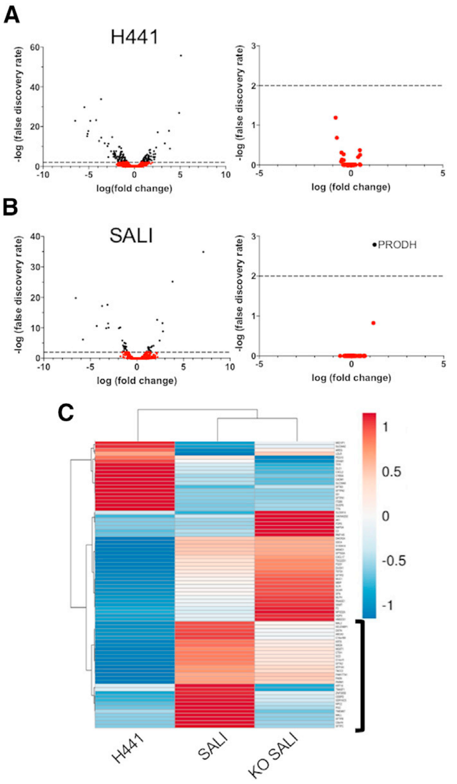

Figure 3. SFTPB KO of SALI culture results in targeted downregulation of surfactant metabolism, tight junction, and other related genes

Volcano plots comparing the differential gene expression in *SFTPB* KO versus WT (A) submerged H441 cells and (B) SALI cultures of (left) all detected genes and (right) predicted off-targets of the guide RNAs used to create the *SFTPB* KO. Differentially expressed genes with false discovery rates less than 0.01 (using Benjamini-Hochberg adjusted p values) are represented with black dots. The dotted lines indicate –log (false discovery rate) = 2. (C) Heatmap analysis comparing the expression levels of the top 100 genes identified in primary human ATII cells. Genes that are related to the surfactant metabolism are indicated with a black bracket. Heatmap was made using ClustVis.[17]

Importantly, *ABCA3*, one of the major markers for pulmonary lamellar bodies (a key structural component of ATII cells where surfactant is stored) was also upregulated (Figures S3C and S4). Overall, these findings imply that the transcription of genes involved in the surfactant metabolism is highly active in SALI cultures, resulting in increased efficiency of surfactant protein production,[11] which is involved in modulating biophysical and structural properties at the air-liquid interface relating to surface tension and functional barrier properties.[16]

SFTPB KO of SALI cultures results in targeted disruption of genes relating to the surfactant metabolism

The transcriptional changes that occur in SPB-deficient ATII cells are largely unknown due to the rarity of the disorder, as well as a general lack of such studies, but the *SFTPB* KO SALI model allowed us to investigate this. Expression levels of a total of 164 (74 up, 90 down) and 48 (22 up, 26 down) genes were significantly altered ($p < 0.01$) in *SFTPB* KO submerged H441 cells and SALI cultures, respectively, compared with their WT counterparts (Figures 3A and 3B). Furthermore, differential expression analysis of predicted off-target genes for the guide RNAs used to create the *SFTPB* KO revealed minimal change in gene regulation, suggesting that KO-related transcriptomic changes in the cells were specific, rather than due to off-target effects of the CRISPR-Cas9 system. Importantly, the KO of *SFTPB* resulted in an overall downregulation of the surfactant metabolism pathway. This was also evident from the three-way gene set enrichment analysis of the top 100 ATII-specific genes (Figure 3C).

Downregulation of surfactant metabolism was also evident in the expression levels of key ATII cell markers as well as proteins involved in the storage and secretion of pulmonary surfactant (Figure 4). In WT SALI cultures, surfactant, specifically SPB, is stored in lamellar body-like intracellular vesicles and secreted into the apical compartment as well as intercellular spaces (Figure 4A). As expected, following the KO, a clear reduction in expression of *SFTPB* and *SFTPC* was observed. Interestingly, *SFTPD* expression was further upregulated, possibly indicative of compensatory biophysical mechanisms to partially retain surfactant functionality (Figure 4B). Importantly, we confirmed the reduction of *ABCA3*, *STX2*, and *SNAP23* expression at both the RNA and protein levels (Figures 4C and 4D). While ABCA3 is a key marker of lamellar bodies, the secretory organelles in ATII cells, where the pulmonary surfactant is stored,[18] STX2 (syntaxin-2) and SNAP23 (synaptosome-associated protein 23) are key proteins involved in the formation of lipid rafts, essential for the fusion of lamellar bodies with the plasma membrane.[19,20] Together, these results reveal a targeted disruption in the regulation of the genes involved in the surfactant metabolism in SALI cultures as a result of the KO of *SFTPB*.

Lack of SPB expression in SALI cultures activates the unfolded protein response and enriches ATI cell-like phenotype

We then set out to investigate mechanisms by which loss of SPB could influence cellular mechanisms. SPB expression is known to be required for the correct processing of SPC as well as the genesis of lamellar bodies.[21] We hypothesized that the lack of SPB could result in mis-processing or accumulation of pro-SPC leading to endoplasmic reticulum (ER) stress and unfolded protein response (UPR), a key disease phenotype associated with SPC deficiency.[22] Investigation of key markers via qRT-PCR and immunoblotting revealed upregulation of apoptosis by the UPR in *SFTPB* KO SALI cultures compared with WT cultures (Figures 5B and 5C). Specifically, we observed increased expression of *PERK*, *HSPA1A*, *HSPA5 (BiP)*, and *XBP1* at the RNA level indicating the induction of ER stress via the PERK and IRE1 pathways.[23,24]

Activation of these misfolded protein response pathways is known to promote apoptosis in cells. This is also evident in KO SALI cultures through the modest upregulation of the caspase cascade (*CASP3* and *CASP9*) as well as *BAX* (Figure 5B). The decrease in cell viability could be a cause of the significant reduction in transepithelial

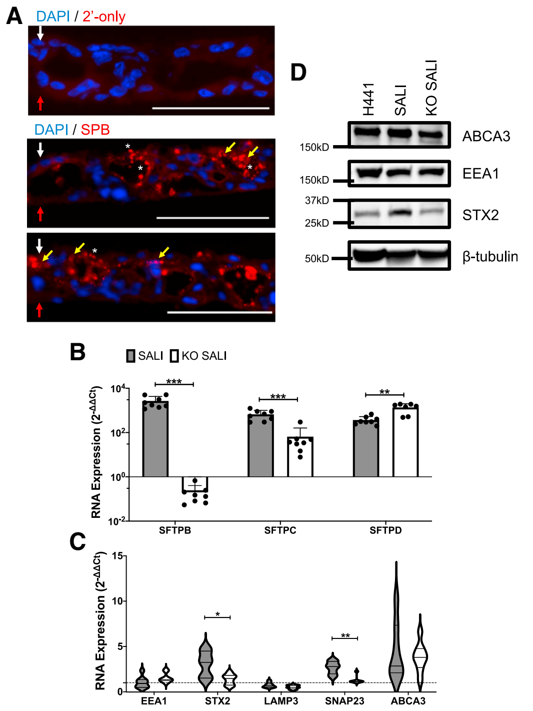

Figure 4. The expression of key ATII cell and lamellar body markers are affected by KO of *SFTPB*

(A) Representative images of surfactant expression in WT SALI cultures. Cross-sections of WT SALI cultures were stained with anti-SPB antibody (n = 4 biological replicates, n = 8 cryosections per replicate) to observe surfactant secretion relative to the apical and basolateral surfaces of the SALI culture (white and red arrows respectively). Surfactant storage in intracellular vesicles (yellow arrows) can be observed, along with surfactant secretion into the apical compartment of the ALI culture (indicated by *), as well as into the intercellular pockets within the SALI model. Nuclei are stained blue. Scale bars represent 100 μm. The expression levels of key (B) ATII cell and (C) lamellar body markers in WT and *SFTPB* KO SALI cultures are summarized in bar graph and violin plot formats, respectively. The RNA expression levels were assessed via qRT-PCR and normalized to that of H441 cells grown in submerged culture using $2^{-\Delta\Delta CT}$ analyses. Each data point represents a biological replicate, columns indicate mean ± SD of data shown. Data shown in the violin plot were acquired from four biological replicates assessed in duplicate. A Mann-Whitney test was performed to compare differences in mRNA levels (*$p < 0.05$, **$p < 0.01$, ***$p < 0.001$). (D) The expression of certain lamellar body markers was also confirmed via immunoblotting. Data shown are representative of three independent repeats. The size of the closest protein marker to each target protein is labeled. *SFTPB*, surfactant protein B; *SFTPC*, surfactant protein C; *SFTPD*, surfactant protein D; *EEA1*, early endosome antigen 1; *STX2*, syntaxin-2; *LAMP3*, lysosome-associated membrane glycoprotein 3; *SNAP23*, synaptosome-associated protein 23; *ABCA3*, ATP binding cassette subfamily A member 3.

electrical resistance we previously observed in KO SALI cultures,[11] suggesting that the disruption of the surfactant metabolism leads to abnormal barrier integrity. This potentially clinically important phenotype is further reinforced by our RNA-seq data, which revealed modest downregulation of several genes for key tight junction proteins, including *TJP1* (encodes zonula occludens 1), *OCLN* (encodes occludin), and *CLDN1*, *3*, *4*, and *7* (encoding claudin-1, -3, -4, and -7) (Figure S5).

Surprisingly, *SFTPB* KO of SALI cultures also led to a statistically significant increase in the mRNA expression of several key ATI cell markers, namely *CAV1* (caveolin-1),[25,26] *AGER* (advanced glycosylation end-product-specific receptor),[26,27] and *HOPX* (homeodomain-only protein homeobox)[26] (Figure 5A). The change in the expression profiles of ATI cell markers, in combination with downregulation of ATII cell markers, implies that the *SFTPB* KO potentially drives SALI cultures to modestly transdifferentiate from an ATII-like phenotype to one which is more akin to that of ATI cells.

DISCUSSION

In vitro cell cultures have the potential to be tractable models to study the human lung alveolar epithelium and related monogenic disorders. Their ability to form reproducible and sustainable long-term cultures makes them prime candidates for the establishment of disease models as well as rapid high-throughput screening assessment of therapeutic interventions. However, they must be physiologically relevant, reflect cellular composition, and parallel gene expression profiles of the primary cells.

We have recently reported a new *in vitro* cell culture platform, SALI, that, unlike similar organoid-based models,[7] successfully recapitulated alveolar cell markers while allowing for long-term, sustainable culture and establishment of relevant disease models. Here, we investigated the transcriptome of the SALI culture platform. Differential gene expression analysis demonstrated that SALI conditions drove significant transcriptional differences in the H441 cells used in the culture model (Figure 1). The main limitation of the SALI culture is that it is based on an adenocarcinoma cell line harboring potentially dysfunctional genomic and transcriptional malignancies.[28,29] Therefore, we compared the transcriptomic changes in H441 cells driven by SALI culture condition to the transcriptional datasets of primary human ATI, ATII, and club cells (Figure 2). We found that the SALI cultures are significantly enriched for ATII cell-specific genes while varying levels of ATI and club cell-specific genes are also expressed (Figure 2).

In a similar fashion, we also observed significant transcriptional changes to *FOXA1* expression between two culture conditions (Figure S4). It has been reported that *FOXA1* plays an important role in epithelial differentiation of lung cells and more importantly negatively regulates transcription of surfactant proteins B and C.[30,31] As *FOXA1* expression decreased, the genes involved in the surfactant metabolism pathway were strongly upregulated in SALI cultures, including auxiliary genes involved in processing, storage, and transportation of SPB- and SPC-based pulmonary surfactant (Figure S2 and S3). The striking similarity between the gene expression profiles of our platform and primary ATII cells highlighted the potential of the SALI culture to serve as a clinically relevant proxy for study of human alveolar cells.

RNA-seq analysis of the *SFTPB* KO SALI cultures revealed not only targeted disruption of *SFTPB* expression but also transcriptional changes in a total of 48 (22 up, 26 down) genes, the majority of which

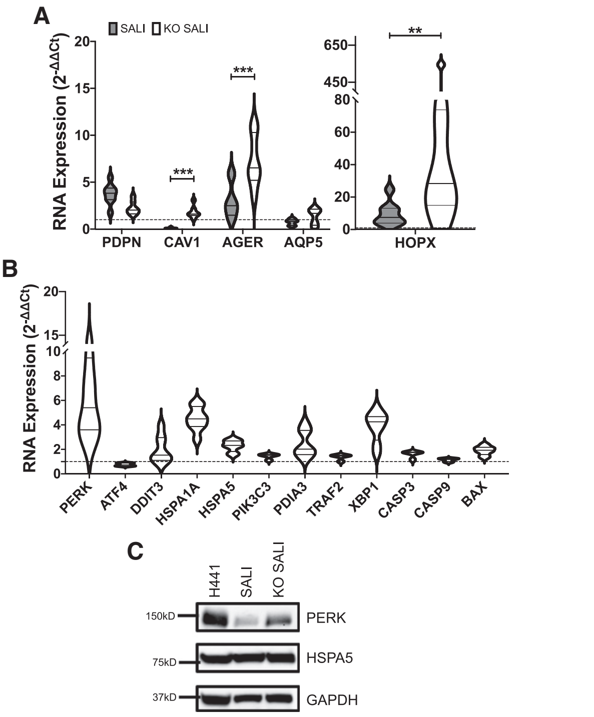

Figure 5. *SFTPB* KO activates unfolded protein response and drives SALI cultures into a more ATI-like phenotype

The expression levels of key endoplasmic reticulum (ER) stress, unfolded protein response, and apoptosis markers were interrogated via (A) qRT-PCR and (B) immunoblotting. The RNA expression levels in *SFTPB* KO SALIs were determined (from four biological replicates assessed in duplicate) and normalized to that of WT SALI cultures using $2^{-\Delta\Delta CT}$ analyses. Immunoblotting data shown are representative of three independent repeats. The size of the closest protein marker to each target protein is labeled. (C) The expression levels of key ATI cell markers in WT and *SFTPB* KO SALI cultures summarized in a violin plot. The RNA expression levels were assessed via qRT-PCR and normalized to that of H441 cells grown in submerged culture using $2^{-\Delta\Delta CT}$ analyses. Each qRT-PCR experiment was performed on four biological replicates in duplicate. A Mann-Whitney test was performed to compare differences in mRNA levels (**$p < 0.01$, ***$p < 0.001$). *PERK*, PKR-like ER kinase; *ATF4*, activating transcription factor 4; *DDIT3*, DNA damage-inducible transcript 3; *HSPA1A*, heat shock protein family A member 1A; *HSPA5*, heat shock protein family A member 5/*BiP*; *PIK3C3*, phosphatidylinositol 3-kinase catalytic subunit type; *PDIA3*, protein disulfide-isomerase A3; *TRAF2*, TNF receptor-associated factor 2; *XBP1*, X-box binding protein 1; *CASP3*, caspase-3; *CASP9*, caspase-9; *BAX*, Bcl-2-associated X protein; *PDPN*, podoplanin; *CAV1*, caveolin-1; *AGER*, advanced glycosylation end-product-specific receptor; *HOPX*, homeodomain-only protein homeobox.

are related to the surfactant metabolism pathway (Figure 3). We confirmed, via qRT-PCR and immunoblotting, that the expression of several key proteins involved in the storage and secretion of pulmonary surfactant, namely STX2, SNAP23, and ABCA3, was disrupted in *SFTPB* KO SALI cultures (Figure 4). The loss of SPB not only affected the surfactant metabolism pathway but also activated the UPR in the SALI cultures (Figures 5A and 5B). The modest upregulation of several key markers, including *PERK*, *HSPA4*, *XBP1*, *CASP3*, and *BAX*, at the RNA level indicated induction of ER stress-related apoptosis pathways in the cells. We speculate that this UPR could stem from abnormalities in lamellar body genesis and mis-processing of SPC, both of which heavily rely on SPB expression.[21]

Interestingly, we observed the upregulation of several ATI cell markers in SALI cultures as a secondary response to the KO of *SFTPB* (Figure 5C). The statistically significant increase in expression of *CAV1*, *AGER*, and *HOPX* in KO compared with WT SALI cultures indicates that the disruption of surfactant metabolism resulted in modest a shift of the SALI model to an ATI cell-like phenotype. Indeed, there have been several studies highlighting similar ATII-to-ATI transdifferentiation in culture following loss of expression of surfactant-associated proteins due to injury or controlled culture conditions.[32–36]

Despite significant potential for interrogating genetics of pulmonary diseases, all *in vitro* models tend to be limited in the ability to recapitulate complex cellular interplay and the environmental features of the lung. Although based on the H441 adenocarcinoma cell line, the transcriptomic analysis of the SALI culture platform confirms its potential as a scalable, relevant, and accurate model for interrogating the genetics of lung diseases affecting alveolar cells. In addition to offering several practical advantages over other existing organoid-based models, our study shows that it replicates the ATII cell transcriptome successfully. In contrast, the expression of ATI markers in SALI cultures lies below physiological levels. In addition, most ILDs, such as idiopathic pulmonary fibrosis, involve numerous cell types, including ATII and ATI cells, fibroblasts, and inflammatory cells. Therefore as a monoculture model, the SALI platform falls short of recapitulating the whole lung parenchyma and unable to mimic the interplay of different cell types in the lung. Nevertheless, the findings presented here, and in our previous study,[11] underline the utility of the SALI platform and its ability to replicate the ATII cell-based alveolar epithelial barrier. We anticipate that the SALI culture platform will be a valuable research tool to study and further understand human ATII cell behavior, as well as to investigate disorders that result in remodeling of ATII cells, such as surfactant-associated pulmonary alveolar proteinosis or epithelial-mesenchymal transition.

MATERIALS AND METHODS

Cells used

NCI-H441 (American Type Culture Collection [ATCC], no. HTB-174, referred to as H441) cells were obtained from the ATCC. *SFTPB* KO H441 cells were generated previously.[11] All cells were maintained in RPMI 1640 (Gibco, no. A1049101) medium supplemented with 50 U/mL penicillin, 50 mg/mL streptomycin (Gibco), and 10% FCS (Sigma). All cells were cultured in a humidified environment at 37°C and 5% CO_2. Cell passages 6–18 for H441 cells were used for this study.

Establishment of SALI cultures

SALI cultures were established as described previously.[11] In brief, approximately 1×10^5 cells/well were seeded into 12-well Transwell inserts (Corning) and allowed to attach and proliferate for 48 h. On

day 3, the medium on the apical side of the Transwell chamber was removed to "air-lift" the cells and the medium on the basolateral side of the chamber was replaced with polarization medium comprising RPMI 1640 supplemented with 2 mM L-glutamine, 50 U/mL penicillin, 50 mg/mL streptomycin, 1% insulin-transferrin-selenium (Gibco), 4% FCS, and 1 μM dexamethasone (Sigma). Cells were grown under SALI conditions for 14 days after air lift before experimentation.

RNA-seq

H441 cells and SALI cultures were grown in parallel. Total RNA was extracted from harvested cell pellets using the RNeasy Mini Kit (QIAGEN) according to the manufacturer's instructions. cDNA was prepared using the NEBNext poly(A) mRNA magnetic isolation module (New England Biolabs). A multiplexed cDNA library was prepared using the NEBNext Ultra II DNA Prep kit for Illumina (New England Biolabs) according to manufacturer's instructions. The prepared library was analyzed on the Illumina NextSeq 500 with 150 base pair paired end read lengths.

Bioinformatics analysis

Reads were aligned to the human reference genome (GRCh37) using HISAT2[37] and duplicate reads removed using the Picard "MarkDuplicates" tool (http://broadinstitute.github.io/picard). Reads mapping uniquely to Ensembl-annotated genes were summarized using featureCounts.[38] The raw gene count matrix was imported into the R/BioConductor environment[39,40] for further processing and analysis with the edgeR package.[41,42] Genes with very low expression were excluded based on the following heuristic: to be retained a gene needed to be expressed at the equivalent of 10 reads or more (after normalizing for library size) in at least as many samples as the smallest experimental group considered (in this case, usually four). Multiple testing correction was performed by using edgeR's default Benjamini-Hochburg method for controlling the false discovery rate.

qRT-PCR

RNA was extracted from ALI cultures and submerged H441 cells using a QIAGEN RNeasy Mini Kit (QIAGEN). On-column DNase I digestion was performed to prevent genomic DNA carry-over according to the manufacturer's instructions. qRT-PCR was carried out directly on extracted RNA samples using TaqMan RNA-to-Ct 1-Step Kit (Applied Biosystems) in a QuantStudio7 Flex Real-Time PCR System (Thermo Scientific). The $2^{-\Delta\Delta CT}$ method was utilized to determine relative expression levels of target genes. β-Actin was used as the reference gene. The TaqMan gene expression assays used for each target gene can be found in Table S1.

Immunoblotting

Immunoblotting was used to confirm the expression of several target proteins in SALI cultures and submerged H441 cells as described previously.[11] In brief, cells were suspended in a lysis buffer supplemented with cOmplete mini protease inhibitor cocktail (Roche). Total protein samples (30 mg) were resolved on 8% SDS-acrylamide protein gels and transferred onto 0.45-μm nitrocellulose membranes (Cytiva Lifesciences). Membranes were blocked in 5% blotting-grade blocker (Bio-Rad) in phosphate-buffered saline (PBS) (Sigma) supplemented with 0.1% Tween 20 (Sigma) for 1 h at room temperature. Following incubation with primary antibodies overnight at 4°C and secondary antibody for 1 h at room temperature, bands were visualized using the Clarity Western ECL Substrate kit (Bio-Rad), and images were captured using the iBright FL1500 imaging system (Invitrogen). Table S2 summarizes all the primary and secondary antibodies used in this study.

Immunofluorescence microscopy

Immunofluorescence microscopy was performed on SALI cultures embedded in optimal cutting temperature compound as described previously.[11] In brief, cryosections were permeabilized and blocked in 1% BSA in PBS solution supplemented with 0.1% Triton X-100 for 1 h at room temperature. Following incubation with primary antibody overnight at 4°C and secondary antibody for 1 h at room temperature, sections were mounted on glass slides using ProLong Gold Antifade Mountant with DAPI. Cells were imaged using the EVOS FL Auto 2 Imaging System (Invitrogen), and images were processed and analyzed using ImageJ software (NIH). Table S2 summarizes all the primary and secondary antibodies used in this study.

DATA AVAILABILITY

All sequencing data (Fastq files) are available on Gene Expression Omnibus (GSE171624).

SUPPLEMENTAL INFORMATION

Supplemental information can be found online at https://doi.org/10.1016/j.omtm.2021.11.006.

ACKNOWLEDGMENTS

The authors thank the Wellcome Center for Human Genetics GMS DPhil Program and the Oxford Genomics Center. A.M.M. is supported by a Wellcome Trust Portfolio Grant 110579/Z/15/Z (to D.R.G. and S.C.H.). The study was in part funded by the Wellcome Center for Human Genetics GMS DPhil Program. As this research was funded in whole, or in part, by the Wellcome Trust, for the purpose of open access, the authors have applied a CC BY public copyright license to any Author Accepted Manuscript version arising from this submission.

AUTHOR CONTRIBUTIONS

A.M.M. designed and performed experiments, analyzed the presented data, and prepared the manuscript. B.W. and H.L. performed bioinformatics analyses. F.J. performed experiments. C.M.G., S.C.H., and D.R.G. supervised the study.

DECLARATION OF INTERESTS

The authors declare no competing interests.

REFERENCES

1. Bradley, B., Branley, H.M., Egan, J.J., Greaves, M.S., Hansell, D.M., Harrison, N.K., Hirani, N., Hubbard, R., Lake, F., Millar, A.B., et al. (2008). Interstitial lung disease

guideline: the British Thoracic Society in collaboration with the Thoracic Society of Australia and New Zealand and the Irish Thoracic Society. Thorax *63*. v1–58. https://doi.org/10.1136/thx.2008.101691.

2. Gower, W.A., and Nogee, L.M. (2011). Surfactant dysfunction. Paediatr. Respir. Rev. *12*, 223–229. https://doi.org/10.1016/j.prrv.2011.01.005.
3. Khor, Y.H., Ng, Y., Barnes, H., Goh, N.S.L., McDonald, C.F., and Holland, A.E. (2020). Prognosis of idiopathic pulmonary fibrosis without anti-fibrotic therapy: a systematic review. Eur. Respir. Rev. *29*, 190158. https://doi.org/10.1183/16000617.0158-2019.
4. Newton, C.A., Molyneaux, P.L., and Oldham, J.M. (2018). Clinical genetics in interstitial lung disease. Front Med. (Lausanne) *5*, 116. https://doi.org/10.3389/fmed.2018.00116.
5. Bove, P.F., Dang, H., Cheluvaraju, C., Jones, L.C., Liu, X., O'Neal, W.K., Randell, S.H., Schlegel, R., and Boucher, R.C. (2014). Breaking the in vitro alveolar type II cell proliferation barrier while retaining ion transport properties. Am. J. Respir. Cell Mol. Biol. *50*, 767–776. https://doi.org/10.1165/rcmb.2013-0071OC.
6. Dobbs, L.G., and Johnson, M.D. (2007). Alveolar epithelial transport in the adult lung. Respir. Physiol. Neurobiol. *159*, 283–300. https://doi.org/10.1016/j.resp.2007.06.011.
7. Barkauskas, C.E., Chung, M.I., Fioret, B., Gao, X., Katsura, H., and Hogan, B.L. (2017). Lung organoids: current uses and future promise. Development *144*, 986–997. https://doi.org/10.1242/dev.140103.
8. Huang, S.X., Islam, M.N., O'Neill, J., Hu, Z., Yang, Y.G., Chen, Y.W., Mumau, M., Green, M.D., Vunjak-Novakovic, G., Bhattacharya, J., et al. (2014). Efficient generation of lung and airway epithelial cells from human pluripotent stem cells. Nat. Biotechnol. *32*, 84–91. https://doi.org/10.1038/nbt.2754.
9. Chen, Y.W., Huang, S.X., de Carvalho, A., Ho, S.H., Islam, M.N., Volpi, S., Notarangelo, L.D., Ciancanelli, M., Casanova, J.L., Bhattacharya, J., et al. (2017). A three-dimensional model of human lung development and disease from pluripotent stem cells. Nat. Cell Biol. *19*, 542–549. https://doi.org/10.1038/ncb3510.
10. Jacob, A., Vedaie, M., Roberts, D.A., Thomas, D.C., Villacorta-Martin, C., Alysandratos, K.D., Hawkins, F., and Kotton, D.N. (2019). Derivation of self-renewing lung alveolar epithelial type II cells from human pluripotent stem cells. Nat. Protoc. *14*, 3303–3332. https://doi.org/10.1038/s41596-019-0220-0.
11. Munis, A.M., Hyde, S.C., and Gill, D.R. (2021). A human surfactant B deficiency air-liquid interface cell culture model suitable for gene therapy applications. Mol. Ther. Methods Clin. Dev. *20*, 237–246. https://doi.org/10.1016/j.omtm.2020.11.013.
12. Reyfman, P.A., Walter, J.M., Joshi, N., Anekalla, K.R., McQuattie-Pimentel, A.C., Chiu, S., Fernandez, R., Akbarpour, M., Chen, C.I., Ren, Z., et al. (2019). Single-cell transcriptomic analysis of human lung provides insights into the pathobiology of pulmonary fibrosis. Am. J. Respir. Crit. Care Med. *199*, 1517–1536. https://doi.org/10.1164/rccm.201712-2410OC.
13. Gazdar, A.F., Linnoila, R.I., Kurita, Y., Oie, H.K., Mulshine, J.L., Clark, J.C., and Whitsett, J.A. (1990). Peripheral airway cell differentiation in human lung cancer cell lines. Cancer Res. *50*, 5481–5487.
14. Rehan, V.K., Torday, J.S., Peleg, S., Gennaro, L., Vouros, P., Padbury, J., Rao, D.S., and Reddy, G.S. (2002). 1alpha,25-Dihydroxy-3-epi-vitamin D3, a natural metabolite of 1alpha,25-dihydroxy vitamin D3: production and biological activity studies in pulmonary alveolar type II cells. Mol. Genet. Metab. *76*, 46–56. https://doi.org/10.1016/s1096-7192(02)00022-7.
15. Sporty, J.L., Horalkova, L., and Ehrhardt, C. (2008). In vitro cell culture models for the assessment of pulmonary drug disposition. Expert Opin. Drug Metab. Toxicol. *4*, 333–345. https://doi.org/10.1517/17425255.4.4.333.
16. Han, S., and Mallampalli, R.K. (2015). The role of surfactant in lung disease and host defense against pulmonary infections. Ann. Am. Thorac. Soc. *12*, 765–774. https://doi.org/10.1513/AnnalsATS.201411-507FR.
17. Metsalu, T., and Vilo, J. (2015). ClustVis: a web tool for visualizing clustering of multivariate data using principal component analysis and heatmap. Nucleic Acids Res. *43*, W566–W570. https://doi.org/10.1093/nar/gkv468.
18. Yamano, G., Funahashi, H., Kawanami, O., Zhao, L.X., Ban, N., Uchida, Y., Morohoshi, T., Ogawa, J., Shioda, S., and Inagaki, N. (2001). ABCA3 is a lamellar body membrane protein in human lung alveolar type II cells. FEBS Lett. *508*, 221–225. https://doi.org/10.1016/s0014-5793(01)03056-3.
19. Abonyo, B.O., Gou, D., Wang, P., Narasaraju, T., Wang, Z., and Liu, L. (2004). Syntaxin 2 and SNAP-23 are required for regulated surfactant secretion. Biochemistry *43*, 3499–3506. https://doi.org/10.1021/bi036338y.
20. Chintagari, N.R., Jin, N., Wang, P., Narasaraju, T.A., Chen, J., and Liu, L. (2006). Effect of cholesterol depletion on exocytosis of alveolar type II cells. Am. J. Respir. Cell Mol. Biol. *34*, 677–687. https://doi.org/10.1165/rcmb.2005-0418OC.
21. Weaver, T.E., and Conkright, J.J. (2001). Function of surfactant proteins B and C. Annu. Rev. Physiol. *63*, 555–578. https://doi.org/10.1146/annurev.physiol.63.1.555.
22. Maguire, J.A., Mulugeta, S., and Beers, M.F. (2011). Endoplasmic reticulum stress induced by surfactant protein C BRICHOS mutants promotes proinflammatory signaling by epithelial cells. Am. J. Respir. Cell Mol. Biol. *44*, 404–414. https://doi.org/10.1165/rcmb.2009-0382OC.
23. Adams, C.J., Kopp, M.C., Larburu, N., Nowak, P.R., and Ali, M.M.U. (2019). Structure and molecular mechanism of ER stress signaling by the unfolded protein response signal activator IRE1. Front Mol. Biosci. *6*, 11. https://doi.org/10.3389/fmolb.2019.00011.
24. Liu, Z., Lv, Y., Zhao, N., Guan, G., and Wang, J. (2015). Protein kinase R-like ER kinase and its role in endoplasmic reticulum stress-decided cell fate. Cell Death Dis. *6*, e1822. https://doi.org/10.1038/cddis.2015.183.
25. McElroy, M.C., and Kasper, M. (2004). The use of alveolar epithelial type I cell-selective markers to investigate lung injury and repair. Eur. Respir. J. *24*, 664–673. https://doi.org/10.1183/09031936.04.00096003.
26. Lamers, M.M., van der Vaart, J., Knoops, K., Riesebosch, S., Breugem, T.I., Mykytyn, A.Z., Beumer, J., Schipper, D., Bezstarosti, K., Koopman, C.D., et al. (2021). An organoid-derived bronchioalveolar model for SARS-CoV-2 infection of human alveolar type II-like cells. EMBO J. *40*, e105912. https://doi.org/10.15252/embj.2020105912.
27. Horie, M., Castaldi, A., Sunohara, M., Wang, H., Ji, Y., Liu, Y., Li, F., Wilkinson, T.A., Hung, L., Shen, H., et al. (2020). Integrated single-cell RNA-sequencing analysis of aquaporin 5-expressing mouse lung epithelial cells identifies GPRC5A as a novel validated type I cell surface marker. Cells *9*, 2460. https://doi.org/10.3390/cells9112460.
28. Castellana, G., Castellana, G., Gentile, M., Castellana, R., and Resta, O. (2015). Pulmonary alveolar microlithiasis: review of the 1022 cases reported worldwide. Eur. Respir. Rev. *24*, 607–620. https://doi.org/10.1183/16000617.0036-2015.
29. Forbes, S.A., Bindal, N., Bamford, S., Cole, C., Kok, C.Y., Beare, D., Jia, M., Shepherd, R., Leung, K., Menzies, A., et al. (2011). COSMIC: mining complete cancer genomes in the catalogue of somatic mutations in cancer. Nucleic Acids Res. *39*, D945–D950. https://doi.org/10.1093/nar/gkq929.
30. Costa, R.H., Kalinichenko, V.V., and Lim, L. (2001). Transcription factors in mouse lung development and function. Am. J. Physiol. Lung Cell Mol. Physiol. *280*, L823–L838. https://doi.org/10.1152/ajplung.2001.280.5.L823.
31. Besnard, V., Wert, S.E., Kaestner, K.H., and Whitsett, J.A. (2005). Stage-specific regulation of respiratory epithelial cell differentiation by Foxa1. Am. J. Physiol. Lung Cell Mol. Physiol. *289*, L750–L759. https://doi.org/10.1152/ajplung.00151.2005.
32. Zhao, L., Yee, M., and O'Reilly, M.A. (2013). Transdifferentiation of alveolar epithelial type II to type I cells is controlled by opposing TGF-beta and BMP signaling. Am. J. Physiol. Lung Cell Mol. Physiol. *305*, L409–L418. https://doi.org/10.1152/ajplung.00032.2013.
33. Ota, C., Ng-Blichfeldt, J.P., Korfei, M., Alsafadi, H.N., Lehmann, M., Skronska-Wasek, W., De Santis, M.M., Guenther, A., Wagner, D.E., and Konigshoff, M. (2018). Dynamic expression of HOPX in alveolar epithelial cells reflects injury and repair during the progression of pulmonary fibrosis. Sci. Rep. *8*, 12983. https://doi.org/10.1038/s41598-018-31214-x.
34. McElroy, M.C., and Kasper, M. (2004). The use of alveolar epithelial type I cell-selective markers to investigate lung injury and repair. Eur. Respir. J. *24*, 664–673. https://doi.org/10.1183/09031936.04.00096003.
35. Jansing, N.L., McClendon, J., Henson, P.M., Tuder, R.M., Hyde, D.M., and Zemans, R.L. (2017). Unbiased quantitation of alveolar type II to alveolar type I cell transdifferentiation during repair after lung injury in mice. Am. J. Respir. Cell Mol. *57*, 519–526. https://doi.org/10.1165/rcmb.2017-0037MA.
36. Kondo, H., Miyoshi, K., Sakiyama, S., Tangoku, A., and Noma, T. (2015). Differential regulation of gene expression of alveolar epithelial cell markers in human lung adenocarcinoma-derived A549 clones. Stem Cells Int. *2015*, 165867. https://doi.org/10.1155/2015/165867.

37. Kim, D., Langmead, B., and Salzberg, S.L. (2015). HISAT: a fast spliced aligner with low memory requirements. Nat. Methods *12*, 357–360. https://doi.org/10.1038/nmeth.3317.

38. Liao, Y., Smyth, G.K., and Shi, W. (2014). featureCounts: an efficient general purpose program for assigning sequence reads to genomic features. Bioinformatics *30*, 923–930. https://doi.org/10.1093/bioinformatics/btt656.

39. R CoreTeam (2021). R: a language and environment for statistical computing. https://www.R-project.org/.

40. Huber, W., Carey, V.J., Gentleman, R., Anders, S., Carlson, M., Carvalho, B.S., Bravo, H.C., Davis, S., Gatto, L., Girke, T., et al. (2015). Orchestrating high-throughput genomic analysis with Bioconductor. Nat. Methods *12*, 115–121. https://doi.org/10.1038/nmeth.3252.

41. Robinson, M.D., McCarthy, D.J., and Smyth, G.K. (2010). edgeR: a Bioconductor package for differential expression analysis of digital gene expression data. Bioinformatics *26*, 139–140. https://doi.org/10.1093/bioinformatics/btp616.

42. McCarthy, D.J., Chen, Y., and Smyth, G.K. (2012). Differential expression analysis of multifactor RNA-Seq experiments with respect to biological variation. Nucleic Acids Res. *40*, 4288–4297. https://doi.org/10.1093/nar/gks042.

OMTM, Volume *24*

Supplemental information

RNA-seq analysis of the human surfactant air-liquid interface culture reveals alveolar type II cell-like transcriptome

Altar M. Munis, Benjamin Wright, Frederic Jackson, Helen Lockstone, Stephen C. Hyde, Catherine M. Green, and Deborah R. Gill

Supplementary Materials

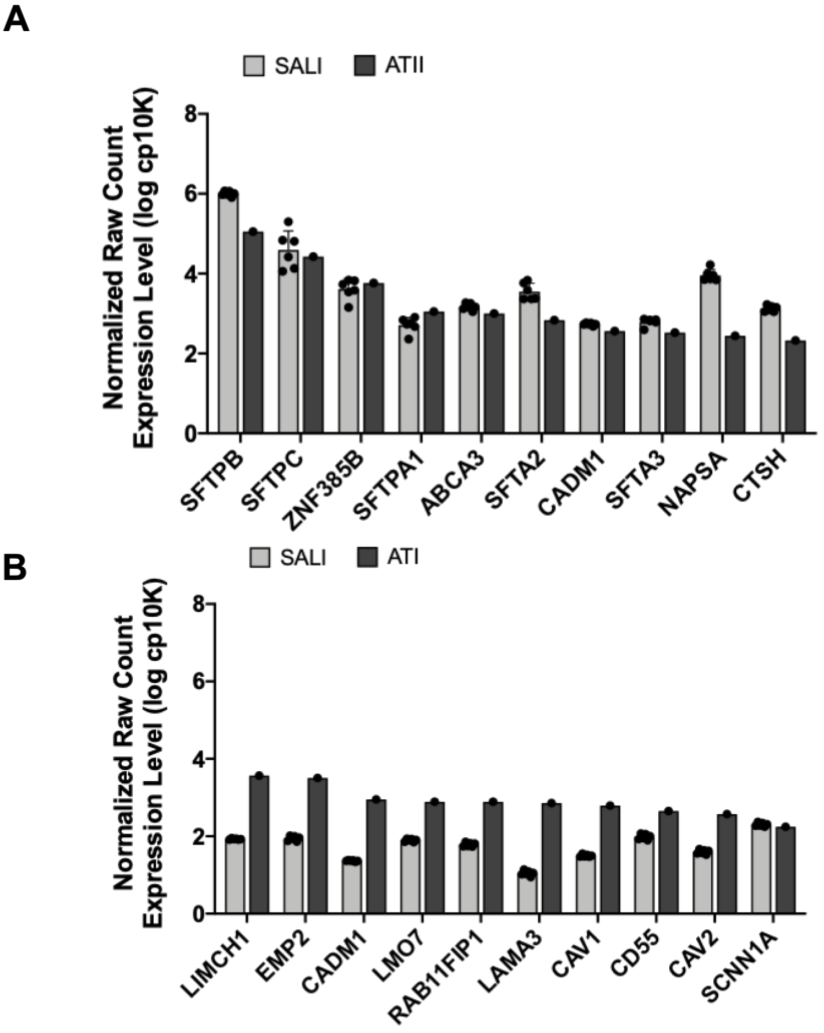

Figure S1: Comparison of the average of normalized count values of select ATII and ATI markers in SALI cultures versus primary cells. Bar graphs depicting the average normalized count values (in log cp10K) of selected **(A)** ATII and **(B)** ATI markers. Each data point represents an individual wt SALI library. The data for the primary human ATII and ATI cells were obtained from the Genotype-Tissue Expression (GTEx) portal on 26/10/2021. The GTEx was supported by the Common Fund of the Office of the Director of the National Institutes of Health, and by NCI, NHGRI, NHLBI, NIDA, NIMH, and NINDS.

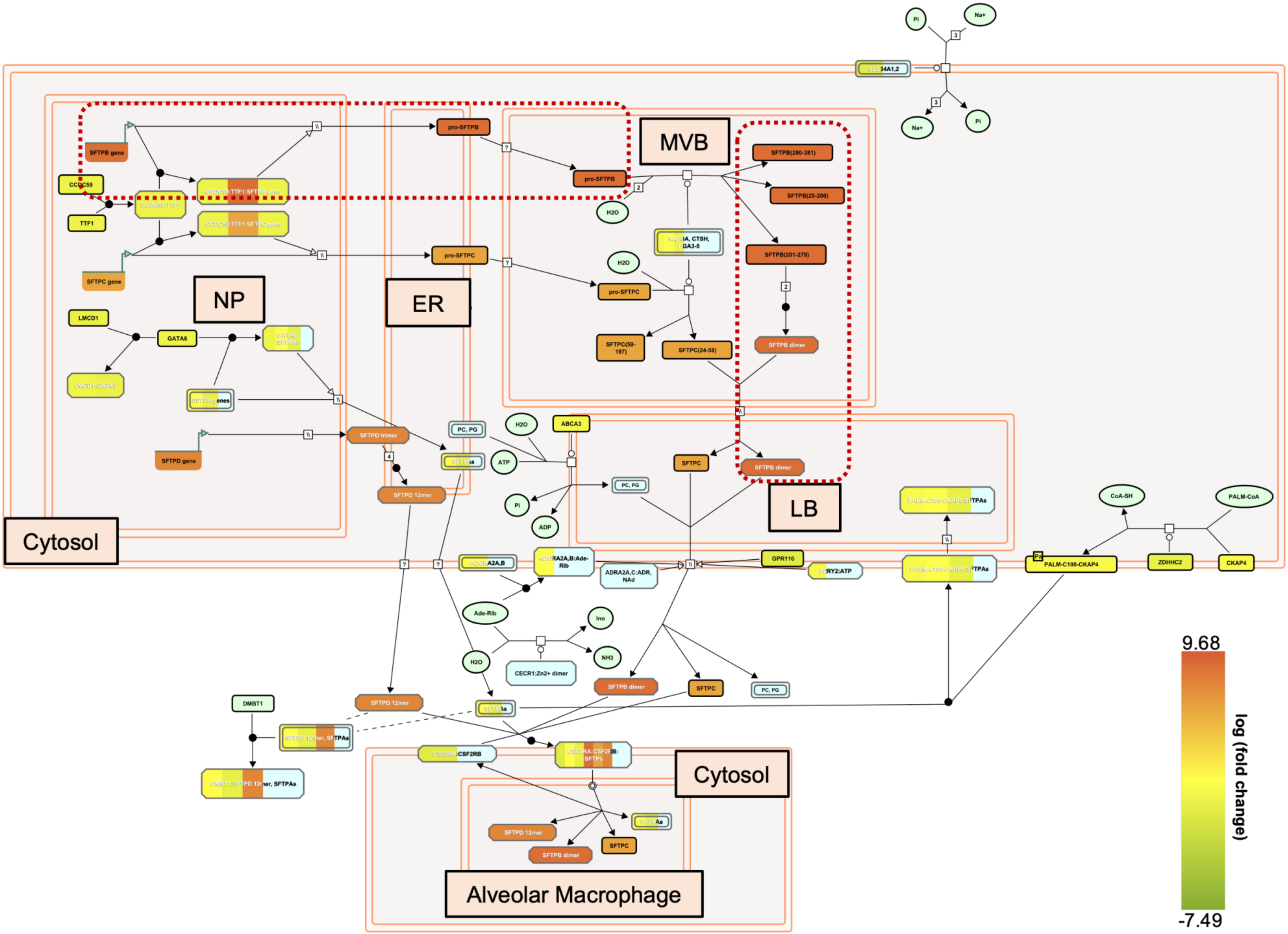

Pi
Na+
3
SLC34A1,2
Na+
Pi
SFTPB gene
CCDC59
TTF1
CCDC59:TTF1
CCDC59:TTF1:SFTPB gene
CCDC59:TTF1:SFTPC gene
SFTPC gene
pro-SFTPB
pro-SFTPC
pro-SFTPB
pro-SFTPC
MVB
H2O
H2O
NAPSA, CTSH, PGA3-5
SFTPB(280-381)
SFTPB(25-200)
SFTPB(201-279)
SFTPB dimer
SFTPC(59-197)
SFTPC(24-58)
NP
ER
LMCD1
GATA6
LMCD1:GATA6
GATA6:SFTPAs
SFTPA genes
SFTPD gene
SFTPD trimer
SFTPD 12mer
PC, PG
SFTPAs
H2O
ATP
ABCA3
Pi
ADP
PC, PG
SFTPC
SFTPB dimer
LB
PALM-C100-CKAP4:SFTPAs
CoA-SH
PALM-CoA
Cytosol
ADORA2A,B
ADORA2A,B:Ade-Rib
ADRA2A,C:ADR, NAd
GPR116
P2RY2:ATP
PALM-C100-CKAP4:SFTPAs
PALM-C100-CKAP4
ZDHHC2
CKAP4
Ade-Rib
Ino
NH3
H2O
CECR1:Zn2+ dimer
SFTPB dimer
SFTPC
PC, PG
DMBT1
SFTPD 12mer
SFTPAs
SFTPD 12mer, SFTPAs
DMBT1:SFTPD 12mer, SFTPAs
CSF2RA:CSF2RB
CSF2RA:CSF2RB:SFTPs
Cytosol
SFTPD 12mer
SFTPAs
SFTPC
SFTPB dimer
Alveolar Macrophage
9.68
log (fold change)
-7.49

Figure S2: A diagram of the surfactant metabolism pathway depicting the relative expression levels of the genes and proteins involved in SALI culture. The differential gene expression data of SALI culture versus submerged H441 cells were analyzed and the results were overlaid on the surfactant metabolism pathway using the open-source pathway database Reactome [1-3]. Regulation of all genes and proteins are labelled and colored based on log (fold change) summarized in the legend. Cellular compartments are labelled. Green circles represent the chemical co-factors that are involved in the pathway. NP: nucleoplasm; ER: endoplasmic reticulum; MVB: multivesicular body; LB: lamellar body; Pi: inorganic phosphate; Na^+: sodium ion; ATP: adenosine triphosphate; ADP: adenosine diphosphate; CoA-SH: coenzyme A; PALM-CoA: Paralemmin-coenzyme A complex; H_2O: water; Ade-Rib: extracellular adenosine; Ino: inosine; NH3: ammonia.

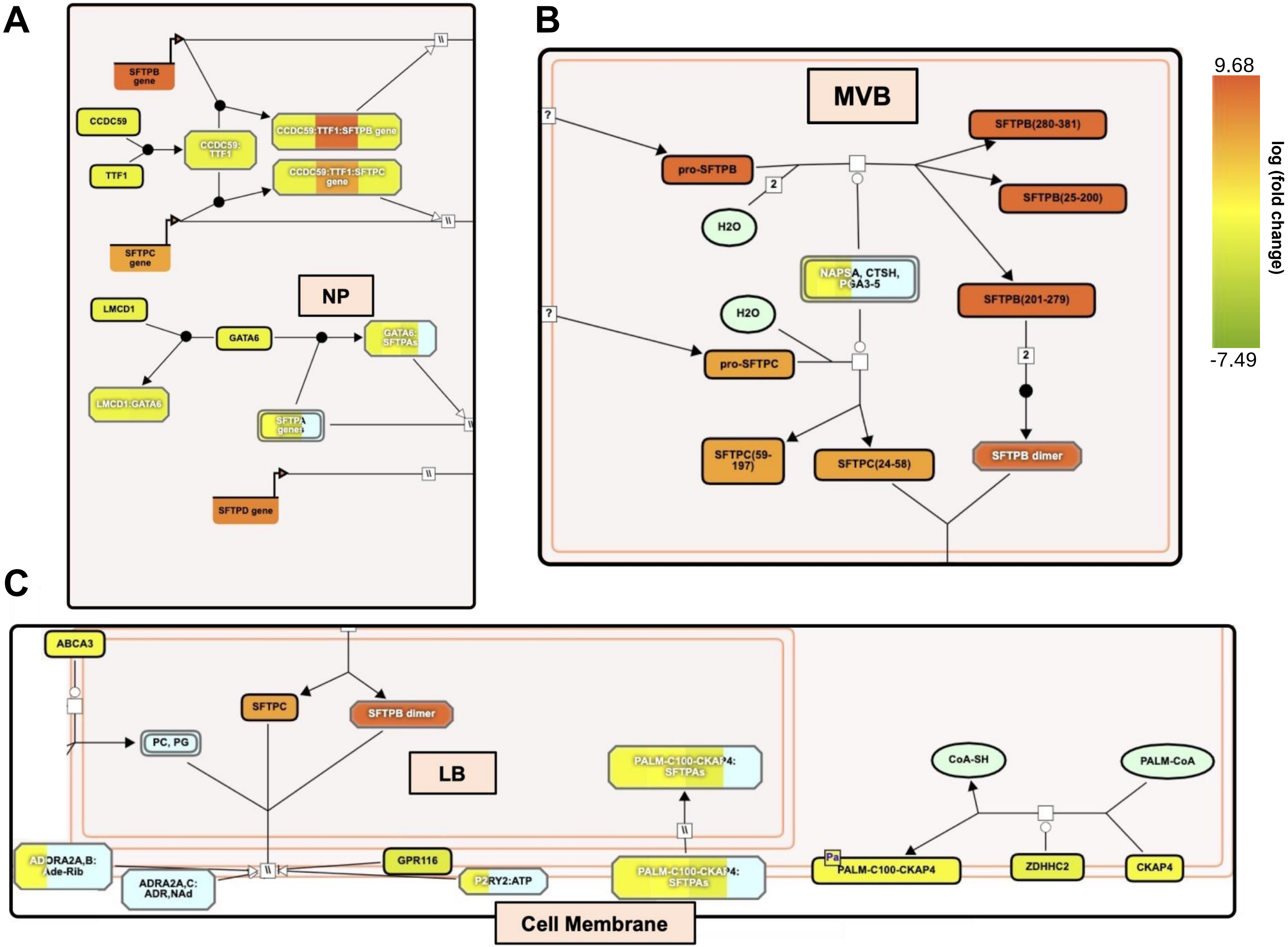
A
SFTPB gene
CCDC59
TTF1
CCDC59:TTF1
CCDC59:TTF1:SFTPB gene
CCDC59:TTF1:SFTPC gene
SFTPC gene
NP
LMCD1
GATA6
GATA6:SFTPAs
LMCD1:GATA6
SFTPA genes
SFTPD gene
B
MVB
?
pro-SFTPB
2
H2O
NAPSA, CTSH, PGA3-5
SFTPB(280-381)
SFTPB(25-200)
SFTPB(201-279)
pro-SFTPC
SFTPC(59-197)
SFTPC(24-58)
SFTPB dimer
9.68
-7.49
log (fold change)
C
ABCA3
PC, PG
SFTPC
SFTPB dimer
LB
PALM-C100-CKAP4:SFTPAs
CoA-SH
PALM-CoA
ADRA2A,B:Ade-Rib
ADRA2A,C:ADR,NAd
GPR116
P2RY2:ATP
PALM-C100-CKAP4
ZDHHC2
CKAP4
Cell Membrane

Figure S3: Genes involved in lung surfactant metabolism are upregulated in SALI cultures. Diagrams of the cellular compartments **(A)** nucleoplasm, **(B)** multivesicular bodies, and **(C)** lamellar bodies depicting the relative expression levels of the surfactant metabolism genes and proteins in SALI culture. The differential gene expression data of SALI culture versus submerged H441 cells were analyzed and the results were overlaid on the surfactant metabolism pathway using the open-source pathway database Reactome [1-3] (See also *Figure S1)*. Regulation of all genes and proteins are labelled and are colored based on log (fold change), depicted in the legend. Cellular compartments are labelled. Green circles indicate the chemical co-factors that are involved in the pathway. Light blue color indicated genes and/or proteins that have not been detected in the SALI culture. NP: nucleoplasm; ER: endoplasmic reticulum; MVB: multivesicular body; LB: lamellar body; Pi: inorganic phosphate; Na^+: sodium ion; ATP: adenosine triphosphate; ADP: adenosine diphosphate; CoA-SH: coenzyme A; PALM-CoA: Paralemmin-coenzyme A complex; H_2O: water; Ade-Rib: extracellular adenosine; Ino: inosine; NH_3: ammonia.

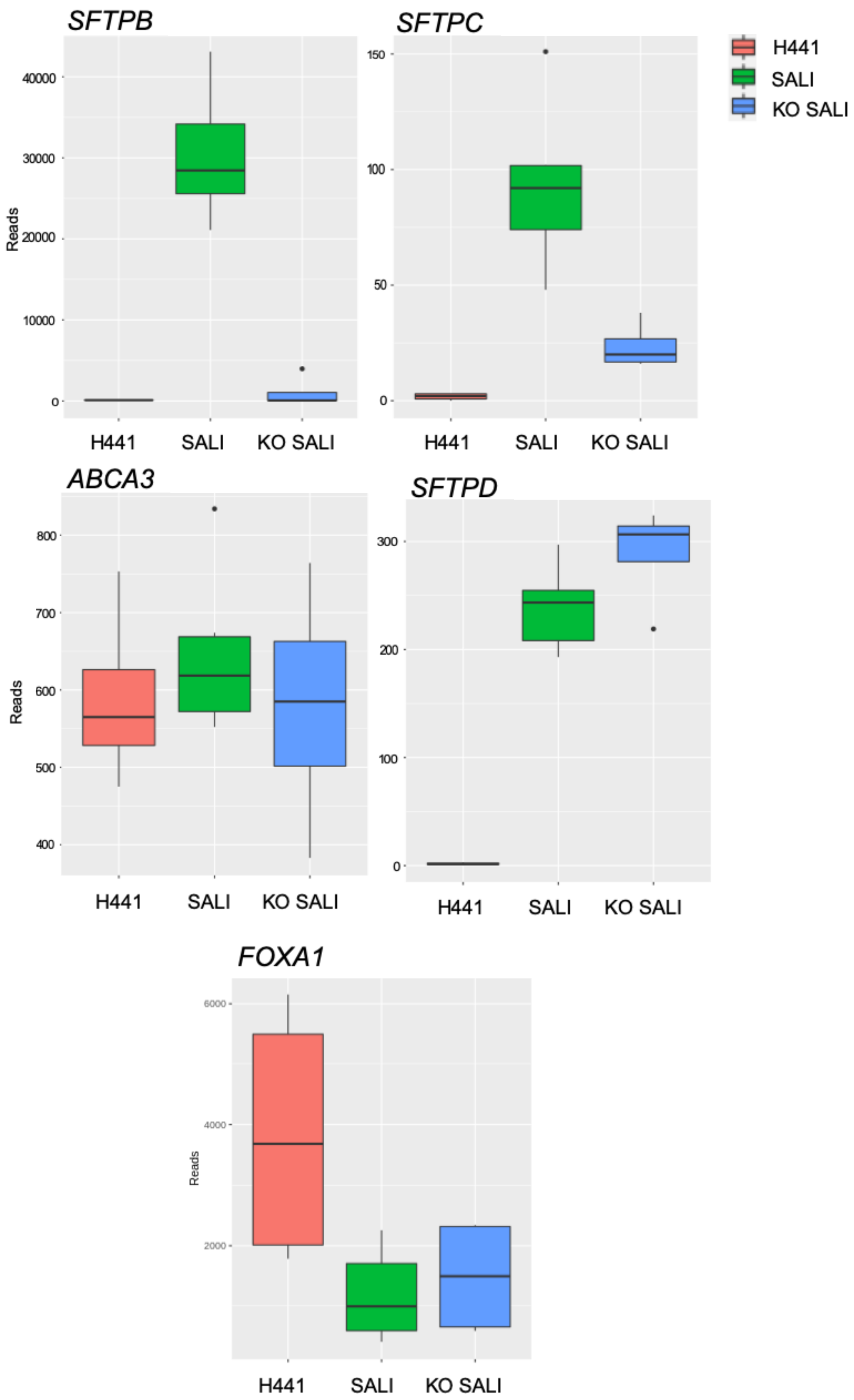

Figure S4: Box and whisker plot depicting the expression levels of *SFTPB*, *SFTPC*, *ABCA3*, *SFTPD,* and *FOXA1* in H441 cells, wild-type and *SFTPB* KO SALI

cultures. Reads were aligned to the human reference genome (GRCh37) using HISAT2[4] and duplicate reads removed using the Picard 'MarkDuplicates' tool (http://broadinstitute.github.io/picard). Reads mapping uniquely to Ensembl-annotated genes were summarized using featureCounts[5]. The raw gene count matrix was imported into the R/BioConductor environment[6,7] for further processing and analysis with the edgeR package[8,9].

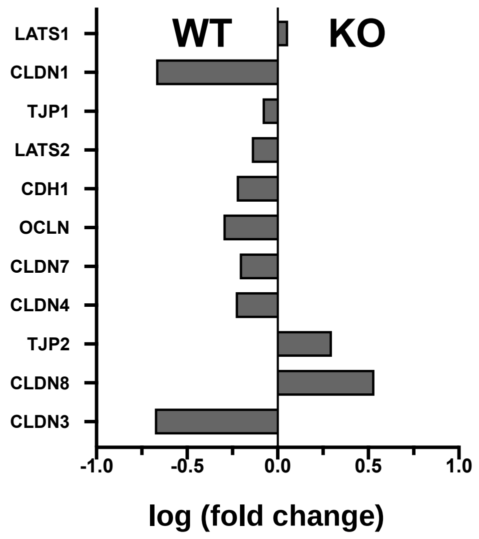

Figure S5: Differential expression analysis of the major tight junction proteins in *SFTPB* KO versus wt SALI cultures. The bar graph represents the mean differential expression of several key tight junction proteins in wt SALI cultures compared with *SFTPB* KO SALI cultures. The log (fold change) values were calculated via the bioinformatics analyses described in the methods section.

Supplementary Table 1: The list of TaqMan gene expression assays used in this study.

**Target**	**TaqMan Gene Expression Assay ThermoFisher ID#**
SFTPB	Hs00167036_m1
SFTPC	Hs00161628_m1
SFTPD	Hs01108490_m1
EEA1	Hs00929215_m1
STX2	Hs00984922_m1
LAMP3	Hs01111316_m1
SNAP23	Hs00187075_m1
ABCA3	Hs00184543_m1
(EIF2AK3) PERK	Hs00984003_m1
ATF4	Hs00909569_g1
DDIT3	Hs01090850_m1
HSPA1A	Hs00359163_s1
HSPA5	Hs99999174_m1
PIK3C3	Hs00176908_m1
PDIA3	Hs00607126_m1
TRAF2	Hs00184192_m1
XBP1	Hs00231936_m1
CASP3	Hs00234387_m1
CASP9	Hs00609647_m1
BAX	Hs00180269_m1
PDPN	Hs00366766_m1
CAV1	Hs00971716_m1
AGER	Hs00153957_m1
HOPX	Hs04188695_m1
ACTB	Hs99999903_m1

Supplementary Table 2: The list of antibodies used in this study.

Target	Antibody	Catalogue Number (Company)
ABCA3	Rabbit anti-ABCA3 polyclonal antibody	#PA5-52478 (Invitrogen)
EEA1	Rabbit anti-EEA1 polyclonal antibody	#ab2900 (Abcam)
STX2	Rabbit anti-STX2 polyclonal antiserum	#110 022 (Synaptic Systems)
β-tubulin	Rabbit anti-beta tubulin polyclonal antibody	#ab6046 (Abcam)
PERK	Rabbit anti-PERK monoclonal antibody	#C33E10 (Cell Signaling Technology)
HSPA5 (BiP)	Rabbit anti- GRP78 BiP polyclonal antibody	#ab21685 (Abcam)
GAPDH	Rabbit anti-GAPDH polyclonal antibody	#G9545 (Sigma)
SPB	Rabbit anti-Pro + mature SPB polyclonal antibody	#ab40876 (Abcam)
Anti-rabbit IgG	Swine anti-rabbit IgG, HRP conjugated	#P0399 (Dako)
Anti-rabbit IgG	Donkey anti-rabbit IgG, Alexa-Fluor 594 conjugated	#A21207 (Invitrogen)

REFERENCES

1. Croft, D., Mundo, A.F., Haw, R., Milacic, M., Weiser, J., Wu, G., Caudy, M., Garapati, P., Gillespie, M., Kamdar, M.R., et al. (2014). The Reactome pathway knowledgebase. Nucleic Acids Res *42*, D472-477. 10.1093/nar/gkt1102.
2. Fabregat, A., Jupe, S., Matthews, L., Sidiropoulos, K., Gillespie, M., Garapati, P., Haw, R., Jassal, B., Korninger, F., May, B., et al. (2018). The Reactome Pathway Knowledgebase. Nucleic Acids Res *46*, D649-D655. 10.1093/nar/gkx1132.
3. Fabregat, A., Sidiropoulos, K., Garapati, P., Gillespie, M., Hausmann, K., Haw, R., Jassal, B., Jupe, S., Korninger, F., McKay, S., et al. (2016). The Reactome pathway Knowledgebase. Nucleic Acids Res *44*, D481-487. 10.1093/nar/gkv1351.
4. Kim, D., Langmead, B., and Salzberg, S.L. (2015). HISAT: a fast spliced aligner with low memory requirements. Nat Methods *12*, 357-360. 10.1038/nmeth.3317.
5. Liao, Y., Smyth, G.K., and Shi, W. (2014). featureCounts: an efficient general purpose program for assigning sequence reads to genomic features. Bioinformatics *30*, 923-930. 10.1093/bioinformatics/btt656.

6. Team, R.C. (2021). R: A language and environment for statistical computing. https://www.R-project.org/.
7. Huber, W., Carey, V.J., Gentleman, R., Anders, S., Carlson, M., Carvalho, B.S., Bravo, H.C., Davis, S., Gatto, L., Girke, T., et al. (2015). Orchestrating high-throughput genomic analysis with Bioconductor. Nat Methods *12*, 115-121. 10.1038/nmeth.3252.
8. Robinson, M.D., McCarthy, D.J., and Smyth, G.K. (2010). edgeR: a Bioconductor package for differential expression analysis of digital gene expression data. Bioinformatics *26*, 139-140. 10.1093/bioinformatics/btp616.
9. McCarthy, D.J., Chen, Y., and Smyth, G.K. (2012). Differential expression analysis of multifactor RNA-Seq experiments with respect to biological variation. Nucleic Acids Res *40*, 4288-4297. 10.1093/nar/gks042.
